# A novel mutation in *NF1* gene of patient with Neurofibromatosis type 1: A case report and functional study

**DOI:** 10.1002/mgg3.1643

**Published:** 2021-03-25

**Authors:** Tingting Zhang, Caiwei Jia, Zhiya Dong, Chuanyin Li, Wenli Lu

**Affiliations:** ^1^ Department of Pediatrics Ruijin Hospital Affiliated to Shanghai Jiao Tong University Shanghai China; ^2^ Cancer Center Shanghai Tenth People’s Hospital School of Medicine Tongji University Shanghai China

**Keywords:** bone maldevelopment, neurofibromatosis type 1, *NF1*, Ras/Erk signaling, short stature

## Abstract

**Background:**

Neurofibromatosis type 1 is an autosomal dominant inherited disease and caused by *NF1* gene mutation. Its clinical manifestations include multiple cafe´‐au lait (CAL) spots, skinfold freckling, neurofibroma, bone dysplasia, learning disabilities, and an increased risk of malignancy.

**Methods and Results:**

Here, we reported a Chinese patient bearing with a novel *NF1* mutation (c.2064delGGATGCAGCGG/p.Gly672AsnfsTer24) and complaining mainly about bone phenotype. Functional studies found that this novel mutation caused the damage of NF1 mRNA and protein levels, and lost the inhibition on Ras/Erk signaling.

**Conclusion:**

A novel mutation in *NF1* gene was identified and *in vitro* functional studies were performed, which provided a potential molecular mechanism to explain the bone maldevelopment of patients with neurofibromatosis type 1.

## Graphical abstract

A Chinese patient bearing with a novel NF1 mutation is involved in this study. The present complaint of this patient is bone phenotype including short stature and scoliosis. Functional studies confirm that this novel mutant could active Ras/Erk signaling and perhaps further inhibit the development of osteoblasts (OBLs), which provide a potential molecular mechanism to explain the bone maldevelopment of patients with neurofibromatosis type 1.

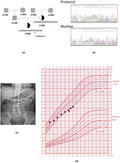

## 1 INTRODUCTION

Neurofibromin, encoded by *NF1* gene, is a GTPase activating protein for RAS with 2818 amino acids. It ubiquitously expresses in multiple organ cells and mostly plays down‐regulating role in RAS‐related signaling pathway (Cichowski & Jacks, 2001; Korf, 2013; Scheffzek & Welti, 2012). The human *NF1* gene is located on chromosome 17q11.2, consisting of 57 exons and four alternatively spliced exons, spanning 282 kb of DNA (Viskochil et al., 1990). Linkage studies showed that loss‐of‐function mutations in the *NF1* gene are causative of Neurofibromatosis type 1(NF1) (OMIM 162200), which is an autosomal dominant inherited disease and characterized by evolving tumors and nontumor manifestations. The most common and histologically benign tumor is neurofibroma, which may affect derm, spine, central nervous system (Lisch nodules in iris mostly) or extend along the nerve and form the plexiform neurofibromas ultimately. 10% adult patients with NF1 have high risks for malignant peripheral nerve sheath tumors (MPNSTs), especially in patients with plexiform neurofibromas (Evans, 2002). Nontumor clinical features of NF1 comprise multiple cafe´‐au lait (CAL) spots, skinfold freckling, skeletal dysplasias, learning disabilities, and vascular dysplasias (Korf, 2013).

Typical skeletal dysplasias contain pseudarthrosis, tibial dysplasia, osteoporosis and scoliosis (Crawford, 1989; Crawford & Bagamery, 1986; Kuorilehto et al., 2004, 2005; Sbihi et al., 1980). Murine models with *NF1* deficit showed similar skeletal abnormity as NF1 patients, including reductive bone mass, tibial bowing and delayed fracture healing (Schindeler et al., 2008; Wang et al., 2010). Besides widely recognized bone characteristics of NF1 described above, short stature (<10th percentile) was also noted. It was reported that short stature has affected ~24% in prepubertal patients and more than 40% at adult height (Carmi et al., 1999). A possible and well‐established mechanism is Ras‐MAPK signaling pathway (Bertola et al., 2005). Skeletal deficits have been considered to be the result of defective osteoclasts (OBLs) differentiation (Schindeler et al., 2008; Wang et al., 2010). Activated Ras/MAPK pathway in NF1 is known to underlie aberrant proliferation and differentiation of OBLs (Yang et al., 2006). Altered extracellular signal‐regulated kinases (Erk1/2) signaling has been found in several genetic diseases with skeletal phenotypes (He et al., 2011). Erk1/2 are phosphorylated and activated by Ras‐Raf‐MEK signaling, then further modulate multiple cell lineages in their proliferation, survival, differentiation, and protein synthesis, including OBLs (Hata et al., 2007; Kono et al., 2007). These experimental evidences support that hyperactive Ras/Erk signaling induced by *NF1* deficiency may negatively regulate OBLs differentiation, which is associated with bone dysplasia in NF1 phenotype.

In this study, the clinical features of a Chinese patient with neurofibromatosis type 1 were described, and gene sequencing discovered a novel mutation in *NF1* gene. Further functional studies verified that this specific *NF1* gene mutation causes hyperactive Ras/Erk signaling, which might participate in impaired process of bone formation.

## 2 MATERIALS AND METHODS

### 2.1 Editorial policies and ethical

A Chinese patient with a novel *NF1* gene mutation was recruited here. This study was approved by the Institutional Review Board of the Ruijin Hospital. The informed consent was obtained from each participant considerations.

### 2.2 Molecular Investigations

DNA was extracted from peripheral blood leukocytes using DNA extraction kit (Qiagen, Hilden, Germany). Custom gene panel was designed and used to capture targeted sequence, covering all exons and flanking sequence (including the 10 bp of introns) of 187 genes which are associated with growth and development of children (Table S1). The procedure for preparation of libraries was consistent with standard operating protocols previously described (An et al., 2019; Dai et al., 2019; Han et al., 2020; Zhang, Chen, et al., 2020). The average mean depth for the targeted regions was 370, and 84.4% of the covered exons had ≥10 reads. Available reads data were 35.1 M. The candidate mutation was confirmed with Sanger sequencing using the following primers: Forward primers: 5’‐CTTGTGAGTTATTGTATGCGGAGAC‐3’; Reverse primers: 5’‐CAGGACATGGCAACCAGAAC‐3’. The reference sequence NM_001042492.3 of *NF1* mRNA was used.

### 2.3 Plasmids construction

The full length of *NF1* cDNA was synthesized by GENEWIZ (Suzhou, China) and inserted into pCDNA3.0 plasmid with N‐terminus flag tagged. Mutation of *NF1* (p. Gly672AsnfsTer24) was introduced by site‐directed mutagenesis as previously reported (Li, Lu, et al., 2020). The shRNAs for *NF1* gene were designed and inserted into pLKO.1 plasmid which was purchased from SIGMA‐ALDRICH (Merck, Germany), and the specific sequences for these shRNAs are provided in Table 1.

**TABLE 1:** Sequences of shRNAs for *NF1*

shRNAs for *NF1*	Target site sequence
scramble	GCGCGATAGCGCTAATAATTT
sh*NF1*‐1	CCATGTTGTAATGCTGCACTT
sh*NF1*‐2	CTTCGAAGCCTTGCCTAAATT
sh*NF1*‐3	CCCAGGGCGCCGGCCCACCCT

### 2.4 Cell culture and transfection

Human HEK293T cell line was kindly provided by Professor Ronggui Hu (Chinese Academy of Sciences, Shanghai, China) and cultured in Dulbecco's modified Eagle medium (DMEM, Life Technologies, USA) supplemented with 10% fetal bovine serum (FBS), 100 U/ml penicillin and 100 mg/ml streptomycin (all from Gibco, Layola, USA) in a 37°C humidified atmosphere of 5% CO_2_. Plasmids were transfected into HEK293T cells using a Lipofectamine 2000 (Life Technologies, Carlsbad, USA) according to the manufacturer's instructions.

### 2.5 Reverse transcription PCR

Total RNA was extracted from cells using a total RNA kit (Tiangen). Complementary DNA (cDNA) was synthesized using ReverTra Ace qPCR RT Master Mix (Toyobo). *NF1* and *GAPDH* were amplified using 2xPCR mixure (Tiangen), and detected by DNA gel electrophoresis as previously reported (Zhang, Zhou, et al., 2020). PCR were performed using the following primers: *NF1*, Forward primers: 5′‐TCAATGCAGTCTTTAGTCGCATTTCT‐3′, Reverse primers: 5′‐GCCAGCAAGAGCTTTTCGTAGAC‐3′; *GAPDH*, Forward primers: 5′‐TATGATTCCACCCATGGCAAATTCC‐3′, Reverse primers: 5′‐CATGAGTCCTTCCACGATACCAAAG‐3′.

### 2.6 Immunoblotting

Immunoblotting was done as previously described (Li, Han, et al., 2020). Briefly, the lysates of HEK293T cells transfected with plasmids were lysed in RIPA buffer (50 mM Tris–HCl (PH 7.6), 150 mM NaCl, 5 mM EDTA, 0.1% sodium dodecyl sulfate (SDS), and 1% NP‐40) supplemented with protease inhibitor cocktails (Roche), subjected to SDS‐PAGE and transferred to a PVDF membrane (Bio‐Rad). The membranes were incubated with the appropriate antibodies against GAPDH (1:5000, 60004–1‐Ig, Proteintech), NF1 (1:500, 27249–1‐AP, Proteintech), Flag (1:1000, 20543–1‐AP, Proteintech), pan‐Ras (1:2000, MABS195, Millipore, Germany), Erk1/2 (1:3000, SAB1305560, Millipore), or phospho‐Erk1/2 (1:500, E7028, Millipore). Secondary antibodies were labeled with HRP, and the signals were visualized using Tanon 5200 Imaging System (Tanon).

### 2.7 Ras‐GTP Assay

Ras‐GTP levels were detected as previously described (Sharma et al., 2013). Briefly, HEK293T cells were lysed in nonionic lysis buffer (20 mM Tris‐Cl PH7.6, 137 mM NaCl, 1 mM EGTA, 1% Triton‐X‐100, 10% glycerol, 1.5 mM MgCl_2_) supplemented with protease inhibitor cocktails, and the Ras activity was determined using a Ras activation assay kit (Millipore). Briefly, GTP‐bound Ras levels were determined by incubating cell lysates with Raf‐1 Ras‐binding domain conjugated to agarose beads followed by an immunoblot using an anti‐pan‐Ras antibody (Millipore) and the total Ras was also detected.

## 3 RESULTS

### 3.1 Identification of *NF1* gene mutation

**FIGURE 1:**
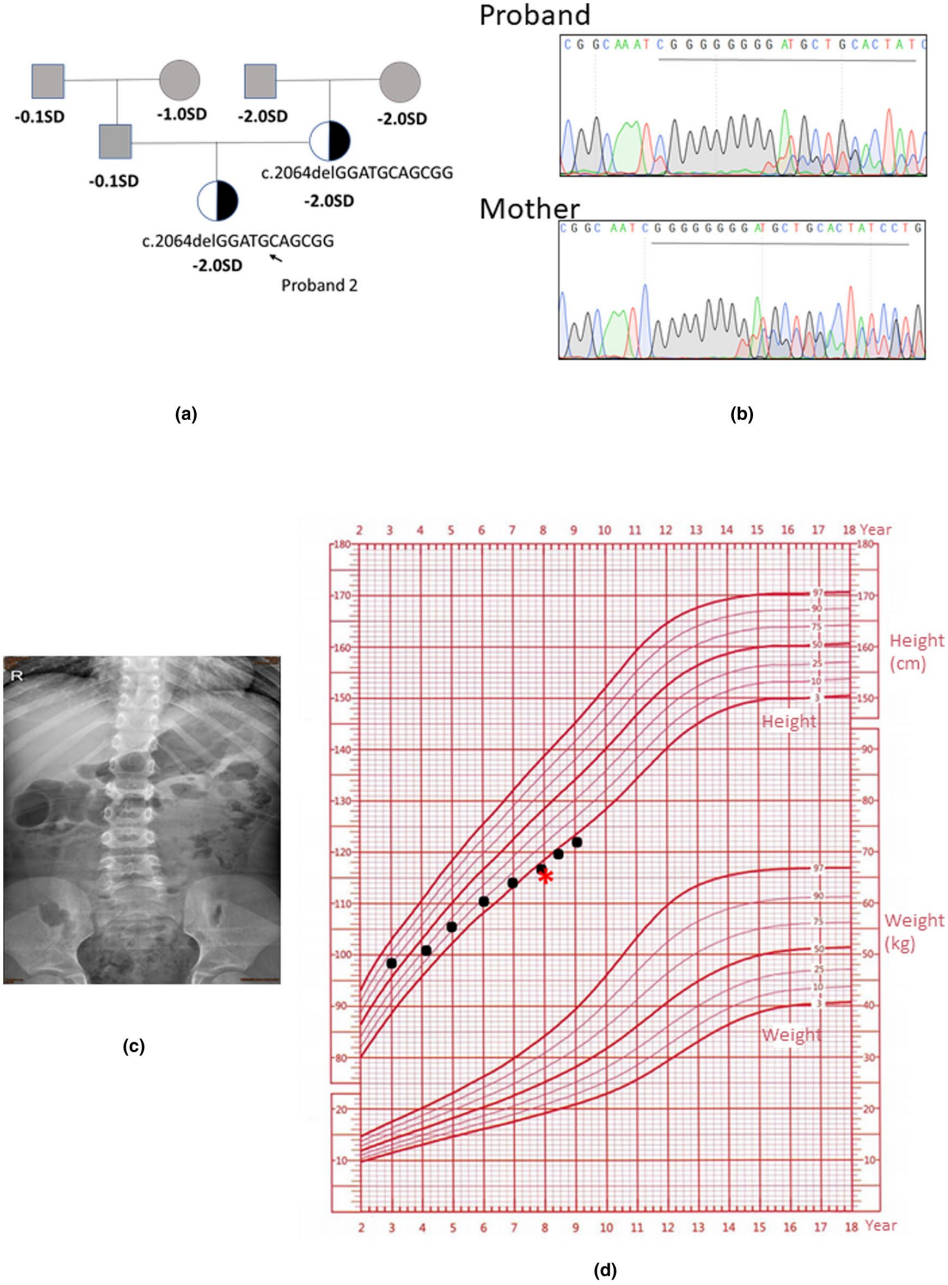
Clinical information of patient. (a,b) Pedigree of the family with a novel c.2064delGGATGCAGCGG mutation in *NF1* gene, and the partial sequencing chromatographs of two family members. (c) X‐ray examination showed a scoliosis of the proband. (d) Growth curves of the proband showed an unideal growth tendency. Black dots refer to patient, red asterisk (*) refers to bone age by Greulich‐Pyle method

Pedigrees of the family with *NF1* gene mutation, and the results of mutation analysis by direct DNA sequencing were shown in Figure1a,b respectively. A frame‐shift mutation (c.2064delGGATGCAGCGG) in exon 18 of the *NF1* gene was found and this mutation introduce a premature stop codon at codon 696 (p. Gly672AsnfsTer24). Although the father was unavailable for testing, the same mutation was found in the proband and her mother.

### 3.2 Clinical characteristics

The affected girl was born at term following an uncomplicated pregnancy and delivery. Her birth weight and length were 3.0 kg and 50 cm respectively. Scattered Café‐au‐lait macules (CALMs) were observed since the child was born. She had an uneventful infancy. At the age of 7 yr and 8 mo, she was admitted to the hospital for short stature (−2.0SD). Physical examination showed pectus excavatum, short fingers, widespread café‐au‐lait spots which were mainly located in trunk. Axillary freckling was notable in both sides. The numbers of CALMs greater than 0.5 cm was six. Symptomatic neurofibroma and subcutaneous nodules were not noted. Slit‐lamp examination did not perform for the patient as she had no complaint of vision abnormity. X‐ray assay revealed scoliosis (Figure 1c) whereas no additional bone abnormality was identified in four limbs. Bone age is equal to chromosome age (by the Greulich‐Pyle method). Ultrasound Bone Sonometers detected an imperfect bone strength in distal radius with Z score as −1.2. Plasma 25‐OHD and 25‐OHD3 were significantly abated. Insulin‐like growth factor 1, thyroid function, and plasma calcium were within normal range. The *NF1* gene mutation was inherited from her mother who showed multiple café‐au‐lait spots, axillary freckling, and a tiny subcutaneous nodule which had been removed by dermatologist 9 years ago. Pathological examination of the nodule revealed a fibroma whereas the documented diagnosis was unavailable. It was said that this subcutaneous nodule firstly appeared on left hip during the mothers’ puberty while no other nodules were developed. The mother also showed an unsatisfied final height of 146 cm (−2.0SD) (Figure 1d **)**.

### 3.3 Mutation of *NF1* cause hyperactive Ras/Erk signaling

**FIGURE 2:**
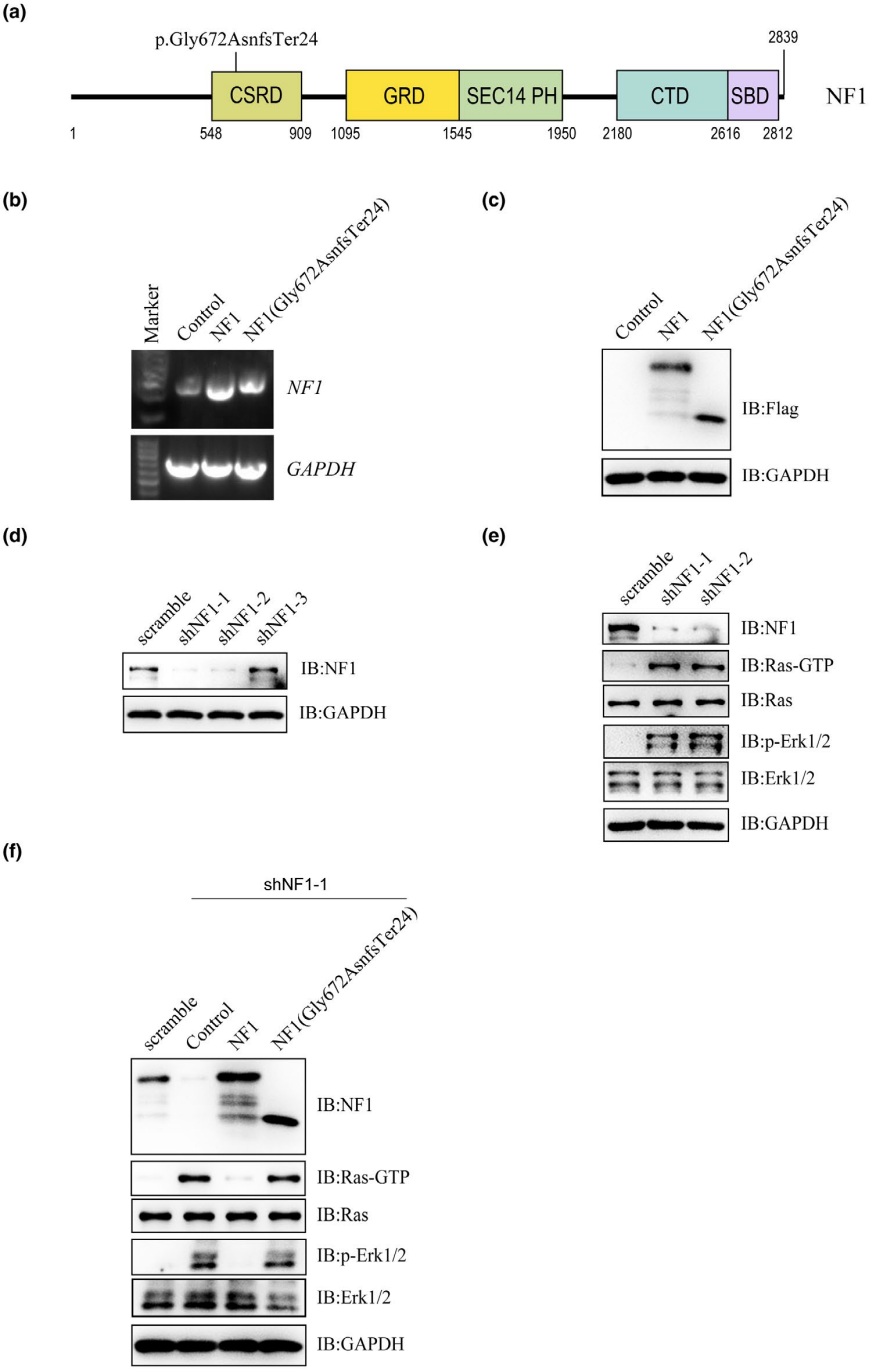
Mutation of *NF1* cause hyperactive Ras/ErK signaling. Schematic view of human NF1 protein mutation involved in this study. CSRD, Cysteine‐Serine‐rich domain; GRD, GTPase‐activating protein‐related domain; SEC14‐PH, SEC14 domain and pleckstrin homology (PH) domain; CTD, Carboxy‐terminal domain; SBD, Syndecan‐binding domain. (a) Detect the mRNA levels of wild‐type and mutant *NF1* by RT‐PCR. Empty vector, wild‐type NF1, or mutant NF1 (p. Gly672AsnfsTer24) were transfected into HEK293 T cells. Total RNA was extracted, complementary DNA (cDNA) was synthesized, and then the mRNA levels of *NF1* and *GAPDH* were detected by PCR. (b) Detect the protein levels of wild‐type and mutant NF1 by immunoblotting. Empty vector, Flag tagged wild‐type NF1 or mutant NF1 (p. Gly672AsnfsTer24) were transfected into HEK293 T cells, and the protein levels of Flag tagged protein and GAPDH were detected by immunoblotting. (c) Test the knockdown efficiency of shRNAs for *NF1* by immunoblotting. HEK293 T cells were transfected with shRNAs (scramble, shNF1‐1, shNF1‐2 or shNF1‐3) for *NF1* gene, and the protein levels of NF1 and GAPDH were detected by immunoblotting. (d) NF1 knockdown activates the Ras‐GTP and phospho‐Erk1/2 signaling pathway. HEK293 T cells were transfected with shRNAs (scramble, shNF1‐1or shNF1‐2) for *NF1* gene, and the indicated protein levels were detected by immunoblotting. (e) Mutation of *NF1* lost the inhibition on Ras/Erk signaling. HEK293 T cells were transfected with shRNAs (scramble or shNF1‐1) for *NF1* gene, as well as transfected with empty vector, Flag tagged wild‐type NF1 or mutant NF1 (p. Gly672AsnfsTer24). The indicated protein levels were detected by immunoblotting

A frameshift mutation of *NF1* gene that expressed a truncated NF1 protein (p. Gly672AsnfsTer24) was involved in this study (Figure 2a). The eukaryotic expression plasmids of wild‐type and mutant *NF1* (p. Gly672AsnfsTer24) were constructed. The mRNA and protein expression of mutant NF1 were lower than that of wild‐type NF1 as revealing by reverse transcription PCR (RT‐PCR) and immunoblotting assays (Figure 2b,c). To study the functions of NF1, three shRNAs targeted the UTR (untranslated region) of *NF1* gene were designed, and the knockdown efficiency were detected by immunoblotting. As showed in Figure 2d, shNF1‐1 and shNF1‐2 exhibited high knockout efficiency, and were selected for further study. *NF1* knockdown significantly activates the Ras‐GTP and phospho‐Erk1/2 signaling in HEK293T cells (Figure 2e). Further study demonstrated that mutation of NF1 lost the inhibition on Ras/Erk signaling compared to that of wild‐type NF1 as detected in *NF1* knockdown HEK293T cells (Figure 2f).

## 4 DISCUSSION

Comparison between several large scale NF1 patient cohorts had emphasized that a large DNA change is more likely to lead a severe phenotype (Rojnueangnit et al., 2015; Theos & Korf, 2006). The truncating protein identified in our patient had missed the GAP‐related domain (GRD), a critical domain that negatively regulates the Ras state (Chen et al., 2005). However, the proband and her mother manifested a mild NF1 phenotype characterized by café‐au‐lait spots and short stature. It was previously observed that phenotypic diversity of NF1 existed in intra‐ and inter‐families and possible explanations include different genetic background, potential modifier factors, mosaicism in affected patients, environmental and stochastic factors (Banerjee et al., 2016; Rojnueangnit et al., 2015). Phenotypes of NF1 would emerge as age progresses (Banerjee et al., 2017). Therefore, we speculate that other NF1‐associated clinical phenotypes may appear in the proband's later life or even do not arise like the asymptomatic condition of her mother.

Growth curve of the proband showed a gradual slowdown in the last 3 years (Figure 1d). Laboratory examinations and lifestyle survey did not find possible explanations. In addition, this patient showed a mild scoliosis. Considering the bone dysplasia in this patient, the substandard final height in her mother and the prevalence of short stature in NF1 patients, we considered the loss‐of‐function variant in *NF1* gene was the rational mechanism responsible for those bone phenotypes.

Besides empirical evidences described in Introduction part, more investigations had exhibited the involvement of Ras/MAPK and Erk/MAPK pathway in bone pathology of NF1 patients. For example, Richa had reported that *Nf1* ^+/−^ osteoprogenitors *in vitro* exhibited a spectrum of bone phenotypes including abnormal proliferation and apoptosis, impaired osteoblast differentiation, and decreased matrix synthesis, which were similar to those of osteoblasts with induced overexpression of Ras (Chen et al., 2005). K Nose *et al*. had proved that increased Ras/MAPK pathway was associated with suppression of c‐fos, whose up‐regulation is required for normal osteoblast functions (Nose et al., 1989). Erks (p44/p42 MAP kinase) were downstream effectors of Ras‐GTP state (Leevers et al., 1994; Stokoe et al., 1994). It was proved that Erk/MAPK pathway plays a negative role in the regulation of type I collagen gene expression in osteoblastic cells (Chaudhary & Avioli, 2000). Shin‐jiroKono with his colleagues had examined the role of Erks in matrix mineralization and finally concluded that Erk pathway is a negative regulator of skeletal mineralization both *in vitro* and *in vivo* (Kono et al., 2007). Richa Sharma *et al*. had reported that phosphorylated Erk1/2 is elevated in *Nf1* ^+/−^ and *Nf1* ^−/−^ osteoblast progenitors (pro‐OBLs) in comparison to wild‐type pro‐OBLs (Sharma et al., 2013).

Consistent with previous findings, our *in vitro* studies did prove that the *NF1* mutant identified from our patient lost the inhibition on Ras/Erk signaling, which could negatively regulate the skeletal growth, providing a more direct evidence to exhibit genotype‐phenotype correlation.

## CONFLICT OF INTEREST

The authors declare that the research was conducted in the absence of any commercial or financial relationships that could be construed as a potential conflict of interest.

## AUTHOR CONTRIBUTIONS

Conceptualization, Wenli Lu and Chuanyin Li; Methodology, Tingting Zhang and Caiwei Jia; Validation, Tingting Zhang and Caiwei Jia; Formal analysis, Tingting Zhang and Caiwei Jia; Investigation, Wenli Lu; Resources, Wenli Lu and Zhiya Dong; Data curation, Tingting Zhang; Writing—original draft preparation, Tingting Zhang; Writing—review and editing, Chuanyin Li and Wenli Lu; Visualization, Chuanyin Lu; Supervision, Zhiya Dong; Project administration, Wenli Lu; Funding acquisition, Wenli Lu. All authors have read and agreed to the published version of the manuscript.

## Supporting information

Table S1

## Data Availability

All datasets generated and analyzed for this study are included in the article.
