# Supplementary material for: A novel mutation in NF1 gene of patient with Neurofibromatosis type 1: A case report and functional study
Source: Mol Genet Genomic Med. 2021 Mar 25;9(5):e1643. doi: 10.1002/mgg3.1643 (PMC8172195; doi:10.1002/mgg3.1643)
Supplement: Supplementary file 1 — Table S1 [file MGG3-9-e1643-s001.docx]

| **growth and development of children** **gene panel for Targeted Next-generation Sequencing.** |
| --- |

| **Function of**  **the genes** | **Genes associated** | | | |
| --- | --- | --- | --- | --- |
| Endocrine growth axis | POU1F1 ，PROP1 ，LHX3 ，LHX4 ，HESX1 ，OTX2 ，PITX ，SOX2 ，SOX3 ，SPR ，  GLI2 ，IGSF1 ，SPINK5 ，GLI3 ，FGF8 ，FGFR1 ，PROKR2 ， HMGA2 ，GRP161 ，  GHRHR ，GH1 ，BTK ，SOX3 ，GHSR ，ALMS1 ，RNPC3 ，IFT172 ，GHR ，STAT5B ，  STAT3 ，IL2RG ， IGF1 ，IGF2 ，IGFALS ，PAPPA2 ，IKBKB ，IGF1R | | | |
| Paracrine factors | FGFR1 ，FGFR2 ，FGFR3 ，GDF5 ，BMPR1B ，BMP2 ，CDMP1 ，ROR2 ，WNT5A ，  PTHLH ，PTH1R ，IHH ，GNAS ，PRKAR1A ，PDE4D ，NPR2 | | | |
| Extracellular matrix | FBN1 ，PAPSS2 ，  MATN3 ，COMP | IDUA ，COL10A1 ，COL9A1 ，COL9A2 ，COL9A3 ，SLC26A2 ，COL2A1 ，ACAN ，ADAMTS10 ，FBN1 | | |
| Intracellular signaling molecules | SHOX ，PTPN11 ，KRAS ，SOS1 ， RAF1 ，NRAS ，BRAF ，RIT1 ，HRAS ，BRAS ，  KRAS ，NF1 ，RPS6KA3 ，FGD1 ，ALMS1 ，SOX9 ，KMT2D ，KDM6A ，TBCE ，FAM111A | | | |
| Syndrome related | CHD7 ，SEMA3E ，SMARCB1 ， | | SMARCA4 ，SMARCA2 ，ARID1A ，ARID1B ， | |
|  | SRCAP ，ANKRD11 ，TRIM37 ， | | PIK3R1 ，POC1A ，CUL7 ， OBSL1 ，CCDC8 ， | |
|  | NIPBL ， SMC1A ， SMC3 ，RAD21 ，HDAC8 ，ORC1 ，ORC4 ，ORC6 ，CDT1 ， | | | |
|  | CDC6 ，U4atac ，PCNT ，TUBGCP6 ，PLK4 ，MECP2 ，CREBBP ，EP300 ，ATR ， | | | |
|  | RBBP8 ，CENPJ ，CEP152 ，CEP63 ，NIN ，DNA2 ，ATPIP ，CRIPT | | | |
| DNA repair defects | RECQL3 ，BLM ，ERCC8 ，ERCC6 ，ERCC3 ，ERCC5 ，ERCC4 ，LMNA ，PCNA ， SMARCAL1 ，MCM4 ，NBS1 ，NBN ，MCM9 ，RECQL4 ，ATRX ，LIG4 ，NHEJ1 ，ARTEMIS ，DNA PKCs ，XRCC4 ，PRKDC ，FANCA | | | |
| Other regulatory factors | IGF2 ，CDKN1C ，THRB ，TRHA ，SECISBP2 ，ESR1 ，VDR ，DHCR7 ，CYP19A1 ， | | | |
|  | INSR ，PITX2 ，CDK6 ，DRD2 ，IGFBP3 ，JAK | | | 2 ，FGF2 ，NPPC ，NPR3 ，COL1A1 ， |
|  | SHOX2 ，MAPK1 ，NFKB1 ，AKT1 ，AKT2 ， | | | GRB2 ，GRB10 ，ZBTB38 ，EHD1 ， |
|  | NBN ，HOXD13 ，MMP13 ，NOG ，RUNX2 ， | | | SLC26A2 ，SOS2 ，TRPV4 |
